# Prevalence and associated risk factors of sarcopenia in community-dwelling older adults in Pakistan: a cross-sectional study

**DOI:** 10.1186/s12877-024-05111-0

**Published:** 2024-06-05

**Authors:** Shafaq Altaf, Kazem Malmir, Syed Mohsen Mir, Gholam Reza Olyaei, Anam Aftab, Tausif Ahmed Rajput

**Affiliations:** 1 Department of Physical Therapy, School of Rehabilitation, Tehran University of Medical Sciences, P. O. Box: 113635 – 1683, Tehran, Iran; 2 Faculty of Pharmaceutical and Allied Health Sciences, Shifa Tameer-e-Millat University, Islamabad, Pakistan; 3 Islam Institute of Rehabilitation Sciences, M. Islam Medical and Dental College, Gujranwala, Pakistan

**Keywords:** Body composition, Community-dwelling, Older adults, Gender, Life expectancy, Pakistan, Prevalence, Sarcopenia

## Abstract

**Background:**

Advancements in medical facilities have led to an increase in global life expectancy, emphasizing the need to address age-related health issues. Sarcopenia, characterized by muscle mass loss, poses significant challenges for older adults. Despite a higher prevalence in Asian populations, there is a remarkable absence of studies addressing sarcopenia among the older adults in Pakistan. This research aims to determine sarcopenia prevalence, identify risk factors, and explore gender- and age-specific patterns among older adults in Pakistan.

**Methods:**

A cross-sectional study involving 142 participants (65 males, 77 females) aged 60 and above was conducted using DEXA scans. Over a six-month period from January to June 2023, data were collected from the Islamabad Diagnostic Centre. This comprehensive dataset covered anthropometric measurements, body composition details, and health parameters. Statistical analyses, including logistic regression, were employed to examine the associations between sarcopenia and various factors.

**Results:**

Sarcopenia manifested in 47.18% of the older adult population (*n* = 142), with a distribution of 39 males (60%) and 28 females (36.36%). The investigation unveiled a compelling correlation between underweight status and sarcopenia across genders. Indeed, males exhibited a significant negative correlation between skeletal muscle mass index and age, whereas females did not show a statistically significant association. Males presented higher odds of sarcopenia in comparison to females (Odds Ratio [OR] = 2.63, 95% Confidence Interval [CI]: 1.33–5.18, *p* = 0.005). Age (OR = 1.12, 95% CI: 1.02–1.22, *p* = 0.014), lower BMI (OR = 0.35, 95% CI: 0.20–0.60, *p* < 0.001), and reduced body fat percentage (OR = 1.75, 95% CI: 1.31–2.33, *p* < 0.001) emerged as significant contributors to sarcopenia. These detailed gender-specific findings emphasize the importance of customizing intervention strategies to address gender disparities in sarcopenia risk factors.

**Conclusion:**

This study highlights the significant prevalence of sarcopenia among older adults in Pakistan, with distinct gender and age-related patterns observed. The overall prevalence of sarcopenia was found to be 47.18%, with higher rates among males compared to females. Age emerged as a significant risk factor, with each additional year increasing the odds of sarcopenia. Furthermore, weight, BMI, lean mass, and total body fat demonstrated important associations with sarcopenia prevalence, highlighting the multifaceted nature of this condition. The practical implications of this study emphasize the need for targeted screening programs and personalized interventions to mitigate sarcopenia’s impact, informing healthcare policies and public health strategies in Pakistan.

## Background

With advancements in medical and treatment facilities, the global average life expectancy has increased from 66.8 years in the early 2000s to 73.4 years in 2019 [1]. The World Health Organization (WHO) predicts that by 2030, around 1 in 6 individuals worldwide will be aged 60 years or older [2]. Aging is consistently linked to a decline in lean body mass, particularly in skeletal muscle and bone mass. The principal components of lean body mass that decline with age include skeletal muscle and bone mass [3]. Skeletal muscle mass and strength start diminishing as early as the fourth decade of life, with about 50% lost by the eighth decade. This decline, constituting around 60% of total body mass, can result in significant challenges for the older adults, impacting their ability to carry out tasks independently [4]. Sarcopenia, characterized primarily by age-related muscle mass loss, may often be accompanied by relative adiposity, and is thus referred to as sarcopenic obesity which has shown to negatively influence the health of the elderly [5]. Furthermore, Sarcopenia leads to reduced body strength, metabolic rate, aerobic capacity, and functional capacity [3] in addition to increase in stress and depression [6]. If left untreated, sarcopenia can impose marked personal, social, and economic burdens, increasing the risk of fractures, impairing routine activities, and contributing to cardiopulmonary diseases and cognitive decline. The financial burden of sarcopenia includes an elevated risk of hospitalization, leading to high care costs [7].

Asian populations exhibit a higher prevalence of sarcopenia compared to other regions. In the older adult population of Asia, the prevalence of sarcopenia, as per the old definition based on low muscle mass, ranged from 6.7 to 56.7% in men and 0.1–33.6% in women [8]. However, with the current definition, which indicates reduced muscle strength and physical performance, the prevalence is observed to be 9.6–22.1% in men and 7.7–21.8% in women [9]. The most frequently adopted definition proposed by the European Working Group on Sarcopenia in Older People (EWGSOP) describes it as reduced muscle mass alone indicating pre-sarcopenia, reduced muscle mass with reduced muscle strength or physical performance as confirmed sarcopenia; while a reduction in both aforementioned parameters in addition to reduced physical performance indicating sarcopenia to be severe [10].

While Dual-energy X-ray absorptiometry (DEXA) is widely recognized for assessing bone density, it also serves to quantify muscle, presenting total body skeletal muscle mass as Appendicular Skeletal Muscle Mass (ASM), or as the muscle cross-sectional area of specific muscle groups or body locations. In contrast, Magnetic Resonance Imaging (MRI) and Computed Tomography (CT) are considered gold standards for non-invasively assessing muscle quantity/mass. However, their utilization in primary care is limited due to elevated equipment costs, lack of portability, and the need for highly trained personnel. Additionally, well-defined cut-off points for low muscle mass are lacking in these modalities. DEXA stands out as a more accessible tool for non-invasively determining muscle quantity, encompassing total body lean tissue mass or appendicular skeletal muscle mass. It is currently favored by some clinicians and researchers for measuring muscle mass. The correlation between muscle mass and body size is fundamental, with individuals of larger body sizes typically having larger muscle mass. Therefore, when quantifying muscle mass, the absolute level of ASM can be adjusted for body size using various methods, such as height squared (ASM/height^2^), weight (ASM/weight), or body mass index (BMI) [11].

The Skeletal Muscle Index (SMI) is calculated as ASM, representing the sum of muscle mass in all four extremities, divided by the height squared. An advantage of DEXA is its ability to provide a reproducible estimate of ASM in a short time when using the same instrument and cut-off points. However, a disadvantage is that DEXA measurements can be influenced by the hydration status of the patient [7]. The cut-off score for SMI indicative of sarcopenia is < 7.25 kg/m^2^ for males and < 5.67 kg/m^2^ for females [11].

Similar to global trends, the population in Pakistan is undergoing an aging process and demographic shift, marked by a rising proportion of older adults. In 2019, Pakistan had 15 million people aged 60 and above, constituting around 7% of the total population [12]. Despite presently representing a smaller percentage, projections indicate a significant surge in the older adult population, reaching an estimated 40 million by 2050 [13]. This demographic shift raises concerns about the potential emergence of sarcopenia as a threat to Pakistan’s geriatric community. The prevalence of sarcopenia in Asia has primarily been documented in East Asian community-dwelling older adults, with rates of 11.5% and 16.7% reported in Japan [14] and 40.3% and 41.3% in South Korea [15] in males and females respectively. A global study also noted a sarcopenic prevalence of 17.5% among older adults in India [16]. Surprisingly, there is a lack of similar studies focusing on Pakistan. Consequently, the primary objectives of the current study are to determine the prevalence of sarcopenia and identify associated risk factors among the older adult population in Pakistan, who are a relatively neglected community with unaddressed issues adding to their chances of hospitalization and overall financial burden on the community. Utilizing DEXA, we aim to assess sarcopenia prevalence, explore gender-specific and age-specific patterns, investigate the relationship with BMI, and examine associations with other health parameters. By accomplishing these objectives, our study aims to offer valuable insights into the prevalence, patterns, and determinants of sarcopenia within the distinctive demographic context of Pakistan. This research contributes to the broader comprehension of age-related muscle mass loss, providing nuanced information that can guide targeted healthcare interventions and public health strategies. These insights hold the potential to shape healthcare practices, offering tailored approaches that align with the unique characteristics of the aging population in Pakistan. Ultimately, our findings aspire to be a cornerstone for informed decision-making, fostering a more effective and responsive healthcare system for the older adult community in the region.

## Methods

### Study design

This investigation utilized a cross-sectional study design to comprehensively investigate the prevalence and influential factors of sarcopenia in the older adult population of Pakistan. Concentrating on individuals aged 60 and beyond, the cross-sectional methodology enabled the concurrent collection of data covering essential anthropometric measurements and details of body composition intricacies. This study provided an in-depth exploration of patterns specific to different age groups, distinctions between genders, and the complex relationship between sarcopenia and BMI.

### Participants

**Fig. 1:**
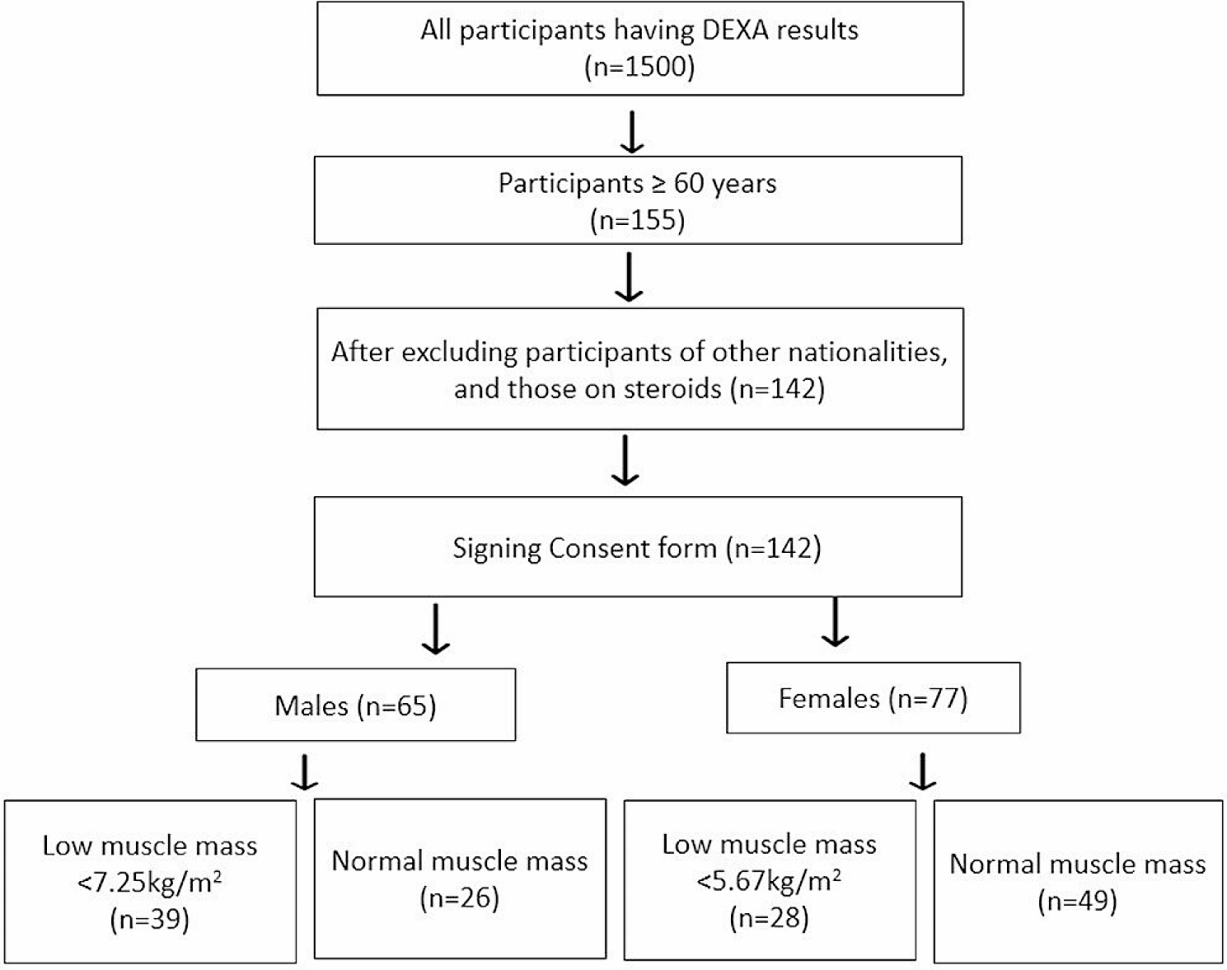
Screening method for including older adults with sarcopenia. Abbreviation: DEXA: Dual-energy X-ray absorptiometry

The data from 142 older adults, encompassing both males (*n* = 65) and females (*n* = 77), who underwent whole-body DEXA, were collected at the Islamabad Diagnostic Centre—a PNAC-licensed laboratory in Islamabad, Pakistan—between January to June 2023. It is essential to note that this cross-sectional study utilized data originally gathered for a registered randomized controlled trial (RCT) investigating the impact of whole-body vibration on SMI in sarcopenic elderly in Pakistan. The RCT, registered with the Iranian Registry of Clinical Trials (IRCT) number # 20230304057612N1, adhered to ethical guidelines, obtaining approval from the ethical board of Tehran University of Medical Sciences (IR.TUMS.FNM.REC.1401.171). Additionally, the protocol has been approved by the Institutional Review Board & Ethics Committee (IRB & EC) of Shifa Tameer-e-Millat University, Islamabad, Pakistan (IRB # 0124 − 23). The ethical aspects, which involve securing informed consent, ensuring participant confidentiality, and safeguarding data, were rigorously adhered to throughout the conduct of the RCT. In this cross-sectional analysis, inclusion/exclusion criteria were established using anonymized and aggregated data from the RCT participants, ensuring alignment with the ethical standards set for the original trial. All participants were aged 60 or above, of Pakistani ethnicity, and had no metal implants including pacemaker in their bodies. Any older adult with a verified frailty status having any severe musculoskeletal disorder and/or on prescribed medications such as steroid therapy known to change body composition, were excluded. Additionally, it was ensured to exclude anyone who could not communicate due to cognitive impairment or severe hearing problems (Fig. 1).

### Estimation of sample size

According to the estimation of sample size for cross-sectional and prevalence studies [17], the sample size is determined by the following formula [18]:

$${\rm{n = }}\left[ {\left( {{{\rm{Z}}^{\rm{2}}}} \right){\rm{P}}\left( {{\rm{P - 1}}} \right)} \right]{\rm{/}}{{\rm{d}}^{\rm{2}}}$$

where n is the sample size, Z represents the confidence level, P is the anticipated prevalence, and d is the precision. Employing an A Priori sample size calculation with a sample proportion within 0.1 of the population proportion, a 95% confidence level, and a precision of 0.25 of P, the calculated sample size is 129.96 participants. Nevertheless, this study included 142 individuals with sarcopenia.

### Anthropometric and body composition measurements

Using a standing scale with a height attachment, participants’ height and weight were measured while they wore light indoor attire and no shoes. Bodyweight was calculated to the nearest 0.1 kg using a balance beam scale with a 0.01 kg margin of error. Height was measured to the closest 0.5 cm while participants stood straight, placing their heads in the Frankfort plane. Based on BMI, participants were categorized as underweight (< 21 kg/m^2^), normal (20 to 24.9 kg/m^2^), overweight (25 to 29.9 kg/m^2^), and obese (> 30 kg/m^2^) [19]. These BMI thresholds approximately align with the classifications suggested by the World Health Organization. To prevent a very small sample size of 4 in the group with a BMI of less than 18.5 kg/m², the boundary separating the underweight and normal weight categories was adjusted from 18.5 to 20 kg/m². Additionally, body composition was assessed using DEXA (Hologic, Discovery Wi). The SMI was determined by summing the skeletal muscle mass for the arms and legs and dividing by height in square meters. The cut-off values for sarcopenic older adults were < 7.25 kg/m^2^ for males and < 5.67 kg/m^2^ for females [11]. Each subject’s scan, conducted by a qualified medical technologist between 10 and 12 A.M, also included assessments of bone health via T-score, lean mass relative to height (kg/m^2^), and total body fat percentage (%). This timing was selected to mitigate the influence of hydration status on DEXA measurements. A T-score from − 1 to -2.5 indicated low bone mass, while < 2.5 denoted osteoporosis. The prevalence of sarcopenia was determined based on gender, age categories, as well as BMI status.

### Statistical analysis

The statistical analysis was conducted using SPSS version 25 with a significance level set at 0.05. Prevalence was described using relative frequencies, with sarcopenia prevalence determined overall and stratified by gender, age, and BMI. Descriptive statistics of numerical variables for both genders were compared using independent sample t-tests or Mann-Whitney U tests. Additionally, Mann-Whitney tests were employed to examine gender differences in sarcopenia prevalence within each BMI category and across various age groups. Simple logistic regression assessed individual impacts of factors (gender, age, weight, BMI) on sarcopenia likelihood, yielding odds ratios (ORs), 95% CIs, and *p*-values. Unadjusted relationships were identified, revealing the individual contributions of these factors to sarcopenia prevalence. To refine the analysis and address potential confounding, multiple logistic regression assessed multiple factors simultaneously, adjusting for covariates. This model provided odds ratios, 95% CIs, and *p*-values for age, gender, weight, and BMI, enhancing comprehension of their independent and combined roles in predicting sarcopenia.

## Results

The descriptive statistics comparing male and female participants in the study are shown in Table 1. Key parameters, including age, height, weight, BMI, T-score, SMI, lean mass per height squared, and total body fat percentage are outlined. The total sample (*n* = 142) had a mean age of 68.06 (6.54) years. Among males (*n* = 65), the mean age was 69.36 (7.12) years, while among females (*n* = 77), the mean age was 66.95 (5.81) years (*p* = 0.038). For height, males (mean = 166.63 (8.29) cm) were significantly taller compared to females (mean = 155.49 (6.14) cm) (*p* < 0.001). No significant differences were observed in weight between males (mean = 72.43 (15.4) kg) and females (mean = 70.71 (12.04) kg) (*p* = 0.458). However, significant differences were found in BMI, with males having a lower mean BMI of 25.92 (4.46) kg/m² compared to females with a mean BMI of 29.13 (5.02) kg/m² (*p* < 0.001). The T-score, representing bone density, averaged − 1.37, indicating lower bone density in the older adults. Interestingly, females showed a slightly lower T-score (-1.48) compared to males (-1.24), suggesting potential gender-specific variations in bone health (*p* = 0.168). Additionally, males tended to have a higher SMI (7.11 kg/m²) than females (6.00 kg/m²) (*p* = 0.001), implying potential gender-related distinctions in muscle composition among the older adult participants examined. Moreover, males had a higher mean Lean Mass (16.62 (2.59) kg/m²) compared to females (15.15 (1.97) kg/m²) (*p* < 0.001), suggesting a greater proportion of lean muscle mass in males. In contrast, females had a higher mean Total Body Fat percentage (46.69 (4.96) %) compared to males (33.50 (5.06) %) (*p* < 0.001), indicating a higher proportion of body fat among females.

**Table 1:** Numerical variables’ descriptive statistics for both genders

Variables	Total (*n* = 142)			Male (*n* = 65)		Female (*n* = 77)		*p* value
	Mean	SD		Mean	SD	Mean	SD	
Age (years)	68.06	6.54		69.36	7.12	66.95	5.81	0.038
Height (cm)	160.60	9.09		166.63	8.29	155.49	6.14	< 0.001
Weight (kg)	71.51	13.67		72.43	15.4	70.71	12.04	0.458
BMI (kg/m^2^)	27.66	5.02		25.92	4.46	29.13	5.02	< 0.001
T-score	-1.37	1.03		-1.24	1.08	-1.48	0.97	0.168
SMI	6.51	1.11		7.11	1.07	6.00	0.86	< 0.001
Lean Mass (kg/m^2^)	15.82	2.39		16.62	2.59	15.15	1.97	< 0.001
Total Body Fat (%)	40.65	8.27		33.50	5.06	46.69	4.96	< 0.001

### Participant characteristics based on age group and gender

The key anthropometric and body composition measures for older adult males and females across distinct age groups are shown in Table 2. The data is derived from 65 older adult males and 77 older adult females. This exploration aims to elucidate age-related trends and potential gender differences in body composition parameters among the older adult population. As can be concluded from Table 2, both males and females exhibit variations in anthropometric and body composition measures across age categories. In males, weight, BMI, and body fat percentage show a decreasing trend with age, while lean mass and SMI exhibit a slight decline. On the other hand, in females, a similar decreasing trend is observed for weight and BMI, while body fat percentage tends to increase with age. Clear distinctions also emerge between males and females within each age group. In general, males tend to have higher values for weight, height, BMI, SMI, and lean mass, while females exhibit higher body fat percentages. In addition, the data suggests that aging may influence body composition differently in males and females. For example, the decline in lean mass and SMI in males is more pronounced compared to females across age groups. The data indicates that, on average, both males and females have T-scores below zero, suggesting a tendency towards lower bone density in this older adult population.

**Table 2:** Participant characteristics based on age group and gender

Gender (*n*) Age group(y)	*n* = 142	Weight (kg)	Height (cm)	BMI (kg/m^2^)	SMI (kg/m^2^)	Lean mass (kg/m^2^)	Body Fat (%)	T-score
Males (*n* = 65)								
60–64	20	77.17 ± 19.1	169.7 ± 9.1	26.57 ± 5.8	7.43 ± 1.3	17.33 ± 3.1	32.6 ± 4.9	-1.2 ± 0.9
65–69	16	73.73 ± 13.4	166.18 ± 8.7	26.62 ± 3.8	7.25 ± 0.9	17.08 ± 1.9	33.9 ± 4.4	-1.31 ± 1.0
70–74	17	68.51 ± 12.1	165.58 ± 6.4	24.87 ± 3.5	6.83 ± 0.9	15.45 ± 2.7	34.2 ± 4.5	-1.45 ± 0.9
≥ 75	12	68.34 ± 14.3	163.62 ± 8.1	25.39 ± 4.0	6.79 ± 0.8	16.50 ± 1.7	33.3 ± 7.0	-0.8 ± 1.5
Females (*n* = 77)								
60–64	31	70.33 ± 12.5	156.09 ± 6.6	28.62 ± 5.5	15.61 ± 2.2	6.06 ± 0.8	45.42 ± 6.1	-1.33 ± 0.8
65–69	23	71.11 ± 13.5	156 ± 5.7	29.22 ± 5.5	15.24 ± 2.3	5.96 ± 1.0	46.8 ± 3.5	-1.60 ± 0.8
70–74	14	72.77 ± 11.1	154.85 ± 5.9	30.27 ± 3.9	14.74 ± 1.2	5.98 ± 0.8	48.38 ± 4.4	-1.51 ± 1.2
≥ 75	9	67.46 ± 8.8	153.05 ± 6.1	28.83 ± 3.6	14.46 ± 1.6	5.86 ± 0.7	46.66 ± 6.7	-1.58 ± 1.2

**SMI**: Skeletal Muscle Index, **T-score**: score equal to or above − 1.0 is considered normal bone density; score between − 1.0 and − 2.5 is considered low bone density, sometimes referred to as osteopenia; score − 2.5 or below is considered osteoporosis

### Trends in sarcopenia prevalence across BMI categories among older adult males and females

**Fig. 2:**
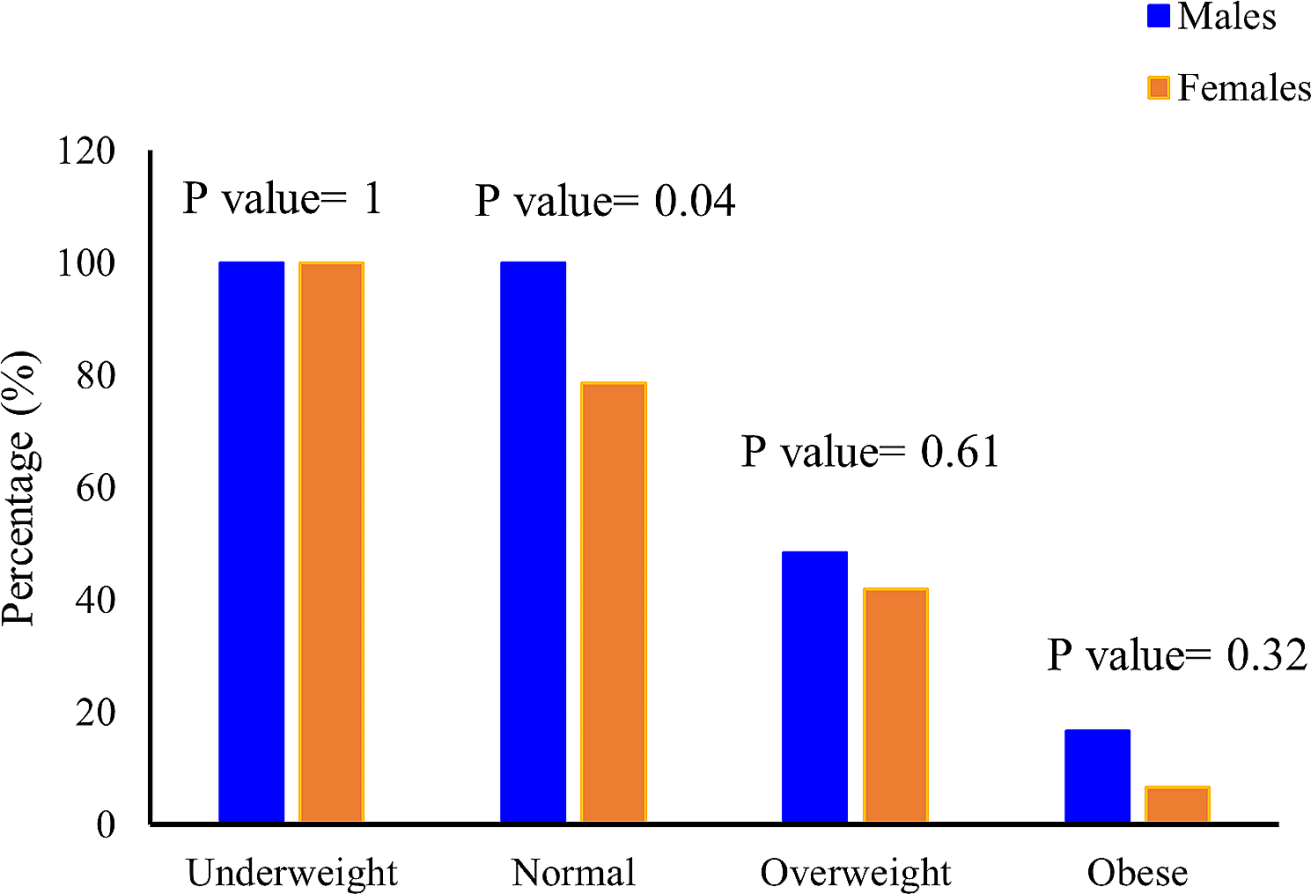
Percentage of sarcopenia among the various BMI categories

Sarcopenia was identified in 47.18% of the older adult population (*n* = 142), with 39 males (60%) and 28 females (36.36%) exhibiting sarcopenia. The prevalence of sarcopenia among various BMI categories is illustrated in Fig. 2. This figure highlights distinctive gender-specific trends. Underweight individuals, regardless of gender, showed a 100% prevalence of sarcopenia, reflecting a significant association between being underweight and sarcopenia. In the normal weight category, all males were affected by sarcopenia, while only 78.57% of females exhibited this condition. Additionally, the prevalence of sarcopenia increased among overweight individuals, with males demonstrating a higher rate than females. Intriguingly, as individuals progressed to the obese category, the prevalence of sarcopenia diminished. Males in the obese category exhibited a 16.67% prevalence, while females showed a lower rate at 6.67%. Mann-Whitney tests were conducted to assess the differences in sarcopenia prevalence between genders within each BMI category. For individuals classified as underweight, no significant difference was found in sarcopenia prevalence between males and females (Z = 0.000, *p* = 1.000). In the normal BMI range, a significant difference appeared, with males revealing a higher prevalence compared to females (Z = -2.031, *p* = 0.042). However, for individuals classified as overweight or obese, no significant differences were observed between genders in sarcopenia prevalence (Overweight: Z = -0.506, *p* = 0.613; Obese: Z = -0.985, *p* = 0.324). These findings suggest that gender differences in sarcopenia prevalence may vary across different BMI categories.

### Age-specific trends in sarcopenia prevalence across genders

**Fig. 3:**
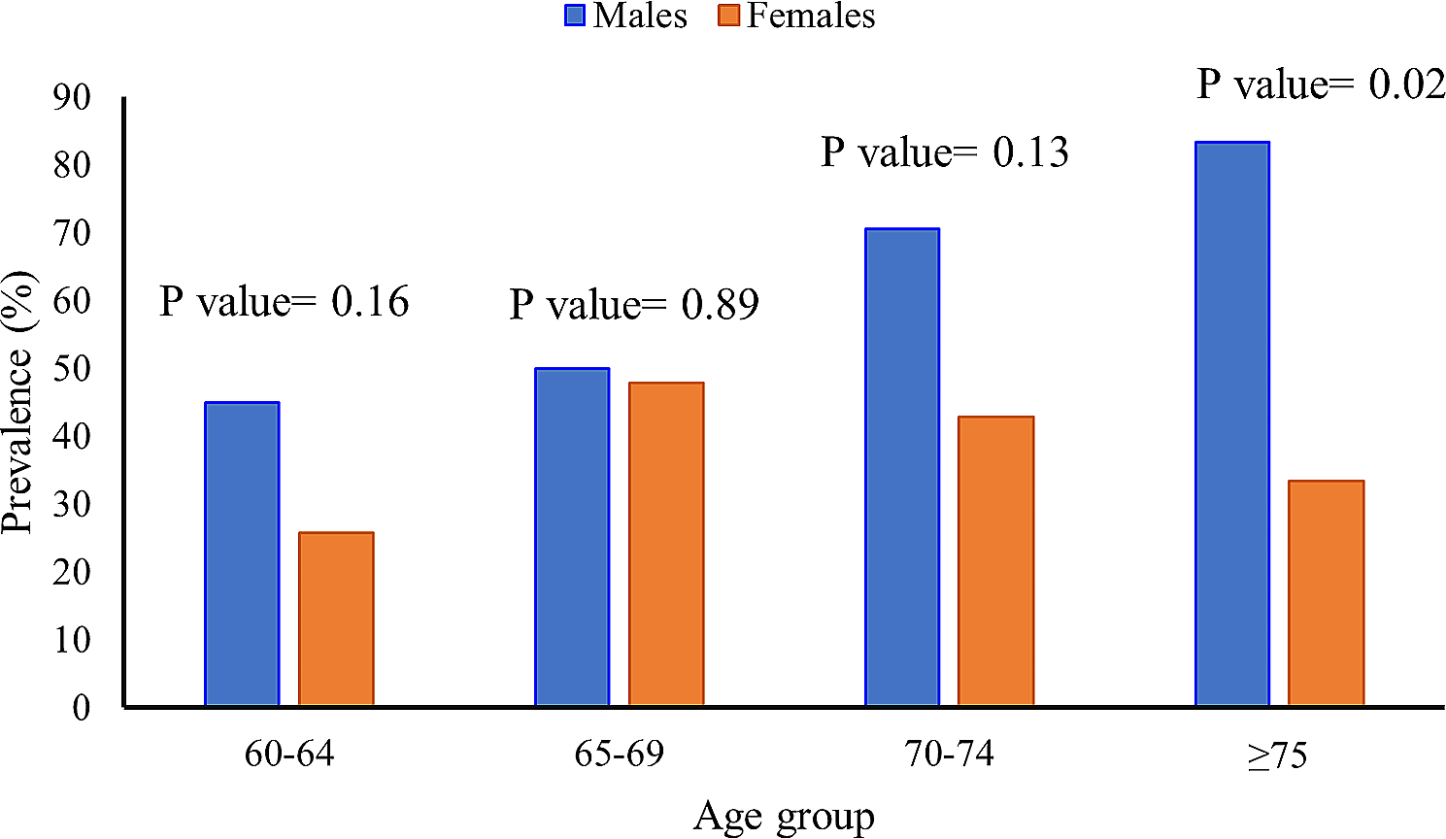
Percentage of sarcopenia among the various age categories

Age-specific and gender-related patterns of sarcopenia prevalence among the older adult population are shown in Fig. 3. Across age groups, a general increase in sarcopenia prevalence is observed, with significant differences between males and females. In the 60–64 age group, males exhibit a higher prevalence (45%) compared to females (25.81%), while in the 65–69 age group, the prevalence becomes comparable (50% in males and 47.83% in females). The trend intensifies in older age groups, reaching the highest prevalence for males (83.33%) in the ≥ 75 age group and for females (33.33%) in the same age category. Mann-Whitney tests were conducted to examine the differences in prevalence of sarcopenia between genders across different age groups. In the age group 60–64, no significant difference was observed between males and females in the prevalence of sarcopenia (Z = -1.406, *p* = 0.160). Similarly, in the age group 65–69, there was no significant difference (Z = -0.132, *p* = 0.895), nor in the 70–74 age group (Z = -1.532, *p* = 0.126). However, among individuals over 75, a significant difference emerged, with females exhibiting a higher prevalence of sarcopenia compared to males (Z = -2.279, *p* = 0.023). These results suggest a gender-related disparity in the prevalence of sarcopenia, particularly among older adults.

### Risk factors for sarcopenia in Pakistani community-dwelling older adults

Table 3 presents the results of both simple and multiple logistic regression analyses investigating the risk factors associated with sarcopenia in Pakistani community-dwelling older adults. In the simple logistic regression model, being male was associated with 2.63 times higher odds of sarcopenia compared to females (OR = 2.63, 95% CI: 1.33–5.18, *p* = 0.005), suggesting a potential gender-based disparity in sarcopenia prevalence. However, the multiple logistic regression model presented a contrasting result, indicating an inverse association between gender and sarcopenia risk, with males demonstrating an exceptionally low odds ratio of 0.001 (95% CI: 0.00-0.036, *p* < 0.001). Age is positively associated with sarcopenia, with each additional year increasing the odds by 1.07 times (OR = 1.07, 95% CI: 1.01–1.13, *p* = 0.014), and this association remains significant in multiple logistic regression (OR = 1.12, 95% CI: 1.02–1.22, *p* = 0.014), albeit with a slightly wider CI (1.02–1.22). Higher weight demonstrates a protective effect, reducing odds by 11% per additional kilogram (OR = 0.89, 95% CI: 0.85–0.92, *p* < 0.001), and this protective trend persists in multiple logistic regression with BMI showing a similar effect (OR = 0.35, 95% CI: 0.20–0.60, *p* < 0.001). Narrow CIs for weight and BMI indicate reliable and precise estimates. Increased lean mass is associated with 0.55 times decrease in odds (OR = 0.55, 95% CI: 0.44–0.69, *p* < 0.001) in simple regression. Meanwhile, higher total body fat is linked to 0.93 times decrease in odds (OR = 0.93, 95% CI: 0.90–0.98, *p* = 0.002), and this association intensifies in multiple logistic regression with a narrower CI (1.31–2.33) remains narrow (OR = 1.75, 95% CI: 1.31–2.33, *p* < 0.001). These detailed results emphasize the multifaceted impacts of gender, age, weight, BMI, lean mass, and total body fat on sarcopenia prevalence, emphasizing the need for a comprehensive understanding of these factors in the context of sarcopenia risk.

**Table 3:** Odds ratios for sarcopenia risk factors

Factors	Simple logistic regression analysis			Multiple logistic regression analysis			
	Odd ratio	95% CI	*p* value	Odd ratio	95% CI	*p* value	
Gender	2.63	1.33–5.18	0.005	0.001	0.00-0.036	< 0.001	
Age (y)	1.07	1.01–1.13	0.014	1.12	1.02–1.22	0.014	
Weight (kg)	0.89	0.85–0.92	< 0.001				
BMI (kg/m^2^)	0.63	0.53–0.73	< 0.001	0.35	0.20–0.60	< 0.001	
Lean mass (kg/m^2^)	0.55	0.44–0.69	< 0.001				
Total body fat (%)	0.93	0.90–0.98	0.002	1.75	1.31–2.33	< 0.001	

## Discussion

The goal of this study was to investigate sarcopenia prevalence in the older adult population of Pakistan and examine variations based on gender, age category, and body composition. Analyzing 142 participants, the study revealed that 47.18% of the older adults were identified with sarcopenia, determined by low muscle mass using DEXA. Despite not measuring grip strength, approximately half of the participants met criteria for being at least “pre-sarcopenic” as per EWGSOP guidelines and confirmed sarcopenic as per EWGSOP2 guidelines, emphasizing the substantial impact on older adults, irrespective of the specific guidelines applied [10].

When considering prevalence rates, it’s noteworthy that prior research in Korea and Indonesia determining SMI via Bioelectrical Impedance Analysis (BIA) and using Asian Working Group on Sarcopenia (AWGS) recommended algorithm, reported sarcopenia in 41% and 41.8% of older adults, respectively [15, 20], figures relatively close to our study’s finding of 47.18%. In contrast, a Japanese study indicated much lower rates, with 11.5% of males and 16.7% of females being sarcopenic [14]. It’s crucial to highlight the methodological differences, as the Japanese study involved a significantly larger sample size (*n* = 1851) and used BIA for muscle mass assessment, unlike our study, which employed DEXA. Speaking of prevalence rates in South Asian population, Xin C et al. (2021) reported the overall prevalence of sarcopenia among the older adults in China as 14% affecting more females than males (15% vs. 14%) [21].

The prevalence of sarcopenia based on gender in our study was, however, higher in men, with 39 individuals (60%) diagnosed, compared to females, where only 28 individuals (36.36%) exhibited sarcopenia. However, the findings of our study contrasts with the results of Reiss et al. (2019), where the number of males diagnosed with sarcopenia, based on SMI via DEXA, was significantly lower, potentially due to differences in muscle strength assessment. Unlike our study, their analysis focused on muscle strength in males, making direct comparison challenging under both EWGSOP and EWGSOP2 guidelines [22]. Similar gender-based prevalence differences were observed by Van Ancum (2020), although the prevalence among females was slightly higher [23]. The use of lower cut-off points for handgrip strength in their study resulted in fewer adults diagnosed with sarcopenia, despite a slightly higher prevalence in females. In contrast to our findings, Shaikh et al. (2020), reported a much higher percentage, with 88.2% of older women in India identified as sarcopenic. Their study used lower hand grip strength cut-offs, potentially encompassing more females [24]. Furthermore, the disparity in our study’s findings may be attributed to the rural setting of the previous study, where a substantial proportion (67.6%) of sarcopenic subjects belonged to below the poverty line. In contrast, the majority of our participants came from a privileged class, able to afford DEXA scans and likely having a relatively better nutritional intake. Additionally, the previous study calculated muscle mass using Lee’s formula [25]. Furthermore, female participants in the current study had a lower SMI than males, which is aligned with previous research. Zhong et al., 2012 reported female groups having significantly lower muscle mass indices compared to male groups. This is probably due to their lower serum testosterone count (TC) and insulin sensitivity (IS). Additionally, most of the cholesterol, which is a precursor of testosterone, comes from endogenous synthesis from unburned food metabolite and thus different cholesterol intake from food may also impose a significant impact on plasma TC and consequently the IS in skeletal muscle [26].

Moreover, our study revealed a distinct age-dependent pattern in sarcopenia prevalence primarily among males, aligning with Huang et al.‘s (2021) findings, which demonstrated a negative correlation between SMI and age in males but no significant association in females [27]. The increased occurrence of sarcopenia in males aged 60–64 highlights a potential gender-specific vulnerability to early muscle mass decline. The prevalence rates aligning in the 65–69 age group indicate a transitional phase where both genders share a comparable vulnerability to sarcopenia. The prominent surge in sarcopenia among males aged ≥ 70 reflects an elevated risk of age-related muscle loss in older males. Conversely, the reduced prevalence in females in the same age group and beyond raises questions about potential protective factors or distinct trajectories of sarcopenia in the oldest female age category. On the other hand, age was associated with sarcopenia in both genders as per a study conducted by Tay et al. (2015) [28]. However, due to the lack of data of physical activity and nutritional intake in the current study, it is impossible to investigate their effects on the differences in SMI between both genders. The findings of the current study emphasize the need for tailored interventions addressing gender-specific vulnerabilities and exploring the complex dynamics of sarcopenia across diverse age groups in the older adult population.

According to our current results, a connection is observed between low BMI and sarcopenia, with the highest prevalence found in this category. Interestingly, within the highest BMI group, particularly among females, the prevalence of sarcopenia is lowest. This contradicts Yoo’s study in 2022, where females in the obese group exhibited significantly lower SMI. It’s important to note that the previous study defined obesity as BMI ≥ 25 kg/m^2^ following the Asia-Pacific criteria, while our study classified obesity as BMI > 30 kg/m^2^ [29].

This study revealed distinct patterns in sarcopenia prevalence among older adults. Simple logistic regression demonstrated that males exhibited higher odds than females, emphasizing a gender-specific vulnerability. However, the multiple logistic regression model yielded a strikingly different result. In this model, males exhibited a low odds ratio, indicating a protective effect against sarcopenia. This discrepancy reflects the complexity of gender’s role in sarcopenia risk. Age was a key factor, with each year contributing to increased sarcopenia risk. Higher weight and BMI offered protective effects, consistently lowering the odds of sarcopenia. Preserving lean mass emerged as crucial in reducing sarcopenia likelihood, highlighting the importance of muscle health. Surprisingly, higher total body fat also showed a protective trend. These associations were robust, supported by narrow confidence intervals, confirming the reliability of our findings. Our study surely illuminates the complex web of factors influencing sarcopenia, offering valuable insights for targeted interventions and healthcare strategies in older population. The results suggest that, considering Pakistan’s cultural norms related to food habits and physical activity, males and older individuals with low body fat and BMI face a higher risk of developing sarcopenia. It’s essential to note that these influencing factors may vary between men and women. The interpretation indicates that specific dietary, cultural, and physical activity traits might impact the identified risk and contribute to the observed patterns. Addressing gender-specific vulnerabilities and age-related patterns, healthcare practitioners can tailor early screening, personalized exercise plans, and nutritional interventions to prevent or alleviate muscle loss. This insight supports the development of educational programs promoting awareness and lifestyle modifications to enhance muscle health. Implementing multidisciplinary approaches that integrate physical activity, nutrition, and regular assessments can effectively minimize the impact of sarcopenia on the aging population in Pakistan, fostering overall health and well-being.

The outcomes of this study accentuate the crucial clinical implications for the health and well-being of the older adult population in Pakistan. The identified prevalence of sarcopenia, marked by distinct vulnerabilities in gender and age-related patterns, poses significant clinical challenges such as compromised mobility, heightened fall risks, and diminished physical functionality. These functional limitations, potentially impacting mental well-being, collectively contribute to a significant decline in the overall quality of life for the older adults. The observed high prevalence of sarcopenia among older adults underscores the urgent need for targeted interventions and public health initiatives aimed at promoting healthy aging and preventing muscle loss. Healthcare practitioners can use these findings to implement early screening programs, develop personalized exercise and nutrition plans, and prioritize muscle health in geriatric care. Furthermore, policymakers can leverage this evidence to advocate for resource allocation towards sarcopenia prevention and management programs, ultimately enhancing the overall well-being and quality of life for older adults in Pakistan.

Future studies seeking a better understanding could overcome some limitations from our research. First, including grip strength measurements, as suggested by EWGSOP2 guidelines, would give us a more complete look at sarcopenia. Also, having larger groups in certain age and BMI categories would make our findings more reliable. Exploring dietary patterns, cultural factors, and levels of physical activity could provide valuable insights for a more comprehensive understanding of sarcopenia. Future studies should prioritize longitudinal research to track the progression of sarcopenia over time in Pakistani older adults. Longitudinal studies can provide invaluable insights into the natural history of sarcopenia, identify risk factors for accelerated muscle loss, and evaluate the effectiveness of interventions aimed at preventing or delaying sarcopenia onset. Additionally, intervention trials should be designed and implemented to assess the efficacy of targeted interventions, including exercise programs, nutritional interventions, and multidisciplinary approaches, tailored to the specific needs of older adults in Pakistan. The practical implications of this study emphasize the need for targeted screening programs and personalized interventions to mitigate sarcopenia’s impact, informing healthcare policies and public health strategies in Pakistan. Future research should focus on longitudinal studies incorporating grip strength measurements and exploring the influence of dietary patterns, cultural factors, and physical activity levels on sarcopenia development.

## Conclusion

This study highlights the significant prevalence of sarcopenia among older adults in Pakistan, with distinct gender and age-related patterns observed. The overall prevalence of sarcopenia was found to be 47.18%, with higher rates among males compared to females. Age emerged as a significant risk factor, with each additional year increasing the odds of sarcopenia. Furthermore, weight, BMI, lean mass, and total body fat demonstrated important associations with sarcopenia prevalence, highlighting the multifaceted nature of this condition.

## Data Availability

The data of the participants are not public but can be made available from the Corresponding Author on reasonable request.

## Abbreviations

ASM: Appendicular Skeletal Muscle Mass
BIA: Bioelectrical Impedance Analysis
BMI: Body Mass Index
DEXA: Dual-energy X-ray absorptiometry
EWGSOP: European Working Group on Sarcopenia in Older People
SMI: Skeletal Muscle Index
